# 

**Published:** 1870-05

**Authors:** 


AMERICAN DENTAL ASSOCIATION—Meets on the first Tuesday in August next year at Nashville, Tenn. *Pres.* H. JUDD, St. Louis. *Rec. Sec.* M. S. DEAN, Chicago.

AMERICAN DENTAL CONVENTION.—Meets in New York, Sept. 1870. *Pres.* Dr. J. G. AMBLER. *Sec.* and *Treas.* Dr. J. H. SMITH, New Haven, Conn.

*List of Societies represented in the American Dental Association.*

AMERICAN DENTAL CONVENTION—Meets annually. *Pres.* J. M. CROWELL of New York, *Rec.* and *Cor. Sec.* J. S. LATIMER, of New York.

AMERICAN ACADEMY OF DENTAL SCIENCE—Meets at Boston, Mass. *Pres.* DANIEL HARWOOD, M. D., of Boston. *Sec.* E. N. HARRIS, D. D. S., of Boston.

BROOKLYN DENTAL SOCIETY—Meets semi-monthly. *Pres.* C. D. Cook. *Rec. Sec.* E L. CHILDS. *Cor. Sec.* WM. JARVIE, Jr.

BUCKS COUNTY DENTAL ASSOCIATION, (PA.) Meets semi-annually on the first Monday in May and November. Next meeting at Newton, in November. *Pres.* H. P. Yerkes, *Sec.* G. W. Adams.

BUFFALO DENTAL SOCIETY—Meets monthly at Buffalo. *Pres.* B. F. Whitney, *Rec. Sec.* S. A. FREEMAN.

BOSTON DENTAL INSTITUTE. Meets first Tuesday of each month. *Pres.* D. G. Williams. *Cor. Sec.* D. S. Dickerman.

CUMBERLAND VALLEY DENTAL SOCIETY—Meets semi-annually.* *Pres.* J. L. SUESSEROTT, of Chambersburg. *Sec.* GEO. W. NEIDICH, of Carlisle, Pa.

CINCINNATI DENTAL SOCIETY—Meets semi-monthly at Cincinnati. *Pres.* Geo. WATT, *Rec. Sec.* Wm. TAFT.

CENTRAL OHIO DENTAL ASSOCIATION—Meets Annually on the second Tuesday of July. *Pres.* S. P. HARRINGTON, Newark. *Sec.* G. W. DECAMP Mansfield, O.

CHICAGO DENTAL SOCIETY—Meets monthly at Chicago. *Pres.* GEO. H. CUSHING. *Rec. and Cor. Sec.* EDGAR D. SWAIN.

CONNECTICUT VALLEY DENTAL ASSOCIATION—Meets semi-annually, second Tuesday of May and October, *Pres.* A. A. HOWLAND, Barre, Vt. *Rec. Sec.* W. H. JONES, of Boston, Mass.

CONNECTICUT STATE DENTAL ASSOCIATION—*Pres.* SAML. MALLETT, New Haven. *Cor. Sec.* JOHN CODY, Hartford. *Rec. Sec.* Dr. POWERS, Hartford.

CHARLESTON DENTAL ASSOCIATION.*—*Prest.* I. B. PATRICK. *Sec. and Treas.* T. F. CHUPEIN.

DELAWARE DENTAL ASSOCIATION—Meets semi-annually. *Pres.* E, W. HAINES, Newark. *Sec.* C. R. JEFFRIES, Wilmington.

EAST TENNESSEE DENTAL ASSOCIATION—Meets annually at Knoxville, Tenn. *Pres.* J. A. CRAZIER, of Knoxville *Rec. Sec.* W. F. FOWLER, Tenn.

FOREST CITY SOCIETY OF DENTAL SURGEONS. *Pres.* B. Strickland, Cleveland. *Rec. Sec.*, H. L. Ambler, Cleveland. *Cor. Sec.*, Chas. Buffett, Cleveland.

GEORGIA STATE DENTAL SOCIETY—Next meeting at Atlanta, July 30th, 1870. *Pres.* F. Y. CLARK; *Cor. Sec.* J. P. H. BROWN, Augusta.

HARRIS DENTAL ASSOCIATION OF LANCASTER, Pa—Next meeting at Columbia, on Thursday the [illegible]th of Nov. next. *President*, SAM'L. WELCHENS. *Sec.* WM. NICHOLS AMER.

HUDSON RIVER ASSOCIATION OF DENTAL SURGEONS—Meets on the second Wednesdays of May, September and January. *Pres.* L. S. STRAW, Newburg N.Y. *Rec. Sec.* T. W. DUBOIS, Pougkeepsie, N. Y.

HUDSON VALLEY DENTAL ASSOCIATION—meets monthly at Troy, N. Y. *Pres.* L. C. WHEELER, *Rec. Sec.* S. G. ANDRES, *Cor. Sec.* S. D. FRENCH.

ILLINOIS STATE DENTAL SOCIETY—Meets annually. *Pres.* M S. Dean, of Chicago. *Sec* C. S. SMITH, of Springfield.

INDIANA STATE DENTAL SOCIETY—Meets annually at Indianapolis on the las Tuesday of June. *Pres.* J. F. Johnston, of Indianapolis. *Rec. and Cor. Sec* S. B Brown.

IOWA STATE DENTAL SOCIETY—Meets annually. *Next meeting at Iowa City, on the second Tuesday of July. *Pres.* J. HARDMAN, of Muscatine. *Rec. Sec.* A. B. MASON, of Waterloo. *Cor. Sec.* J. P. WILSON, of Burlington.

LAKE ERIE DENTAL ASSOCIATION.—Meets Annually.* *Pres.* B.T. WHITNEY, Buffalo N. Y., *Sec.* W. E. MAGILL, Erie, Pa.

LEBANON VALLEY DENTAL ASSOCIATION—*Prest.* W. K. BRENIZER, *Sec.* S. H. GUILFORD.

MAD RIVER VALLEY DENTAL SOCIETY—Meets Semi annually on the first Tuesday of May next. Next meeting at Cincinnati, O. *Pres.* J. H. PAINE, Middletown, O. *Sec.*, S. B. TIZZARD, Troy, O.

MAINE DENTAL SOCIETY—Meets in August, prox., at Biddeford. *Pres.* THOS. HALEY. *Sec.* E. G. ROBERTS.

MARYLAND ASSOCIATION OF DENTISTS—Meets the last Thursday of every month. *Pres.*, J. B. BEAN, *Sec.*, S M. FIELD.

MASSACHUSETTS DENTAL SOCIETY—Meets monthly. *Pres.*, T. H. Chandler, *Rec. Sec.* A. Brown, *Cor. See.* E. Blake.

MICHIGAN DENTAL ASSOCIATION—Meets annually second Tuesday of Oct. at Jackson. *Pres.* E. S. HOLMES. of Grand Rapids; *Rec. and Cor. Sec.* G. H. MOSHER, of Jackson.

MUSKINGUM VALLEY DENTAL ASSOCIATION—Meets on first Monday of each month at Zanesville, O. *Pres.* W. M. Herriot, of Zanesville, O., *Sec.* W. W. Chapelear Zanesville, O.

MISSISSIPPI VALLEY DENTAL ASSOCIATION—Meets annually at Cincinnati, on the first Wednesday in March. *Pres.* W. H. MORGAN, Nashville. *Rec. Sec.* C. M. WRIGHT, Cincinnati, O. *Cor. Sec.* N. W. Williams, Xenia, O.

MISSOURI DENTAL ASSOCIATION—Meets on the first Tuesday of June, in St. Louis. *Pres.* H. JUDD, St. Louis, *Sec.* W. H. EAMES, St. Louis.

MISSOURI VALLEY DENTAL SOCIETY.* Meets on the second Tuesday in July and January. *Pres.* E. J. WOODBURY. of Council Bluffs, Iowa. *Sec. and Treas.* J. F. Sanborn, Tabor, Iowa.

MERRIMAC VALLEY DENTAL SOCIETY—Meets semi annually first Tuesday of Oc and May.* *Pres.* I. H. KIDDER, Mass. *Rec. Sec.* A. M. DUDLEY, of Lowell. *Cor. Sec.* D. T. PORTER, Lawrence, Mass.

MEMPHIS DENTAL SOCIETY.—Meets on the first Tuesday of each month. *Pres* W. T. Arrington, *Sec.* V. F. Elliott. *Treas.* C. L. Harris.

NASHVILLE DENTAL ASSOCIATION—Meets on the second Tuesday of each month *Pres.* J C. Ross, *Sec* R. R. FREEMAN, *Cor. Sec.* S. J. COBB.

NEW YORK STATE DENTAL SOCIETY—Meets at Albany on the last Tuesday of June next. *Pres.* B. T. WHITNEY, of Buffalo. *Sec.* CHAS. BARNS, of Syracuse.

NEW HAVEN DENTAL SOCIETY—Meets on the first Wednesday of each month at New Haven. *Pres.* J. T. METCALF, *Rec. and Cor. Sec.* C. L. SMITH, of New Haven.

NEW LONDON COUNTY DENTAL SOCIETY—Meets monthly.* *Pres.* R. W. BROWNE. *Rec. Sec.* WM. ALLENDER

NORTHERN OHIO DENTAL ASSOCIATION—Meets Semi-annually, (first Tuesday o May and October.) in Cleveland. *Pres.* W. P. HORTON, *Rec. Sec.* H L. AMBLER, *Cor. Sec.* CHAS. BUFFETT, Cleveland.

NORTHERN IOWA DENTAL ASSOCIATION—Meets annually, on the 2d Tuesday in June, 1870. Next meeting at Waterloo. *Pres.* E. S. CLARK, Dubuque. *Rec. Sec.* J. Z. ABBOTT, Manchester. *Cor. Sec.* A. B. MASON, Waterloo.

OHIO DENTAL COLLEGE ASSOCIATION—Meets annually in March, at Cincinnati *Pres.* Geo H. Cushing, Chicago, Ill. *Rec Sec.* J. TAFT, of Cincinnati.

OHIO STATE DENTAL ASSOCIATION.—Meets semi-annually, at Columbus. [Nex meeting—first Tuesday of December.] *Pres.* F. H. REHWINKLE, Chillicothe, *Rec. Sec.* Wm. M. HERRIOTT, Zanesville, O. *Cor. Sec.* A. W. MAXWELL, Galion, O.

ODONTOGRAPHIC SOCIETY OF PENNSYLVANIA—Meets monthly in Philadelphia Next meeting, Wednesday, Sept. 1st. *Pres.* J. H. McQUILLEN. *Rec. Sec.* A. BOICE. *Cor. Sec.* T. C. STELLWAGEN.

ODONTOLOGICAL SOCIETY OF NEW YORK CITY—Meets the second Tuesday of each month.* *Pres.* C. E. FRANCIS. *Rec Sec.* C. F. IVES.

OLD COLONY DENTAL ASSOCIATION—Meets quarterly.* *Pres.* C. G. Davis. *Rec. Sec.* L. W. PUFFER, North Bridgewater, Mass. *Cor. Sec.* N. C. FOWLER, Yarmouth, Mass.

PENNSYLVANIA ASSOCIATION OF DENTAL SURGEONS—Meets monthly in Phila. *Pres.* E. WILLIAMS. *Rec. Sec* JAMES TRUMAN *Cor. Sec.* E. T. DARBY.

PITTSBURG DENTAL ASSOCIATION—Meets first Tuesday of each month in Pittsburg. *Pres.* C. L. WESTENBURG *Rec. Sec.* J. D. WHITE.

SAN FRANCISCO DENTAL ASSOCIATION.—Meets monthly. *Pres.* C. C. KNOWLES *Cor. Sec.* W. J. YOUNGER.

POUGHKEEPSIE DENTAL SOCIETY.—Meets monthly, *Pres.* J. H. Mann, *Sec.* H. F. Clark.

ST. LOUIS DENTAL SOCIETY—Meets monthly. *Pres.* ISAAC COMSTOCK. *Sec.* R. J. POORE

SUSQUEHANNA DENTAL ASSOCIATION—Meets semi-annually*. *Pres.* C. S. BECK M. D. *Rec. Sec.* R. C. BURLEM.

SOCIETY OF DENTAL SURGEONS OF THE CITY OF NEW YORK—Meets semi monthly in New York. *Pres.* B. W FRANKLIN. *Rec. Sec.* J. S. LATIMER. *Cor. Sec* T. H. BURRAS.

SOUTHERN DENTAL ASSOCIATION.—Meets annually.* Next meeting the 2d Wednesday in April, 1870, at Charleston, S. C. *Pres.* JAS. KNAPP. *Rec. Sec.* Prof. ANGELL, New Orleans

STATE DENTAL SOCIETY OF PENNSYLVANIA.—Meets annually Third Tuesday in June.* Next meeting in Pittsburg, 1870. *Pres.* T. L. Buckingham of Philadelphia. *Rec. Sec.* Geo. W. Neidich *Cor. Sec.* Samuel Welchens.

TENNESSEE DENTAL ASSOCIATION.—Meets semi-annually. *Pres.* W. T. Arrington of Memphis *Sec.* Wm M. R. Johns, of Somerville.

TUSCARAWAS VALLEY DENTAL SOCIETY.—The annual meeting in Nov. 1870. *Pres.* JOHN McKINLY, Uhricsville. *Sec.* L. E. DISNEY, Coshocton

WASHINGTON CITY DENTAL ASSOCIATION—Meets on the first Monday of each month. *Pres.* R. F. HUNT. *Sec.* J. C SMITH. *Librarian*, H. N. WADSWORTH.

**Place of meeting fixed by appointment*

### DISTRICT DENTAL SOCIETIES OF THE STATE OF NEW YORK.

*1ST DISTRICT DENTAL SOCIETY.—*Pres.* A. L. Northrop, New York City. *Sec.* O. A. JARVIS, New York City.

2D DISTRICT DENTAL SOCIETY.—*Pres.* W. B. Hurd, Brooklyn. *Rec. Sec.* Wm. Jarvie Jr., Brooklyn. *Cor. Sec.* L. S. Straw, Newburg

3D DISTRICT DENTAL SOCIETY.—Meets at Albany semi-annually and annually. *Pres* J. A. Perkins, Troy. *Sec.* S. J. Andress, of Albany.

*4TH DISTRICT DENTAL SOCIETY.—*Pres.* F. C. Rich, Saratogo Springs. *Sec.* C. A Elmore, Fort Edwards.

*5TH DISTRICT DENTAL SOCIETY.—*Pres.* G. A. Foster, Utica *Sec.* Charles Barnes, Syracuse.

*6TH DISTRICT DENTAL SOCIETY.—*Pres.* S. H. McCall, Binghampton. *Sec.* F. B. DARBY, Elmira.

*7TH DISTRICT DENTAL SOCIETY.—*Pres.* A. G. COLEMAN, Canandagua. *Sec.* H. S. MILLER, Rochester.

8TH DISTRICT DENTAL SOCIETY.—Meets annually on the 1st Tuesday in May, at Buffalo. *Pres.* L. W. BRISTOL, Lockport. *Sec.* S. A. FREEMAN, Buffalo.

THE 7TH AND 8TH DISTRICT SOCIETIES—Hold a union meeting on the first Tuesday of October, alternately at Buffalo and Rochester.

* Place and time of meeting fixed by appointment.

### EXCHANGES,

ST. LOUIS MEDICAL AND SURGICAL JOURNAL,
BOSTON MEDICAL AND SURGICAL JOURNAL,
THE PACIFIC MEDICAL AND SURGICAL JOURNAL, SAN FRANCISCO.
BUFFALO MEDICAL AND SURGICAL JOURNAL,
PHILADELPHIA MEDICAL AND SURGICAL REPORTER.
CINCINNATI LANCET AND OBSERVER,
DENTAL COSMOS, PHILADELPHIA.
L'ART DENTAIRE, PARIS, FRANCE.
BRAITHWAITE'S RETROSPECT, N. Y.
THE DENTAL TIMES, PHILADELPHIA,
LADIES' REPOSITORY, CINCINNATI.
HALL'S JOURNAL OF HEALTH, N. Y,
CHICAGO MEDICAL EXAMINER.
XENIA TORCHLIGHT.
THE DETROIT REVIEW OF MEDICINE AND PHARMACY.
MEDICAL RECORD, N Y.
NASHVILLE JOURNAL OF MEDICINE AND SURGERY,
AMERICAN ECLECTIC MEDICAL REVIEW, N. Y.
AMERICAN JOURNAL OF DENTAL SCIENCE, BALTIMORE.
THE UNITED STATES MEDICAL AND SURGICAL JOURNAL. CHICAGO,
NEW ORLEANS JOURNAL OF MEDICINE.
WESTERN JOURNAL OF MEDICINE. INDIANAPOLIS, IND.
AMERICAN AGRICULTURIST, N. Y.
DENTAL OFFICE AND LABORATORY, PHILA.
BOSTON JOURNAL OF CHEMISTRY.
CINCINNATI MEDICAL REPERTORY.
CHICAGO MEDICAL INVESTIGATOR.
JOURNAL OF APPLIED CHEMISTRY, N. Y.
DRUGGISTS' CIRCULAR AND CHEMICAL GAZETTE, NEW YORK.
DER ZAHNARZT, LEIPZIG.
DEUTSCHE VIERTELJAHRSSCHRIFT, NUREMBURG.
CANADA JOURNAL OF DENTAL SCIENCE, MONTREAL.
GUARDIAN OF HEALTH AND EDUCATION, BOSTON.
HARDWICKE'S SCIENCE GOSSIP, LONDON, ENGLAND.
PACKARD'S MONTHLY, NEW YORK.
POPULAR SCIENCE REVIEW, LONDON, ENGLAND.
MISSOURI DENTAL MONTHLY ST. LOUIS.
MEDICAL BULLETIN, BALTIMORE, MD.
ECLECTIC MEDICAL JOURNAL, CINCINNATI.
TRANSACTIONS, COLLEGE OF PHYSICIANS, PHILADELPHIA.

### The Dental Register

OF THE WEST.

*Issued Monthly, at $3 a Year in Advance.*

DEVOTED TO THE INTERESTS OF THE DENTAL PROFESSION.

EDITED BY J. TAFT AND G. WATT.

The volume commences in January and closes in December.

The Register being issued monthly is always fresh, therefore indispensable to the practitioner who desires to keep posted up with the new inventions and improvements which are daily developed. Neither expense nor effort will be spared to give value and variety to its contents. Articles will be illustrated with well executed engravings whenever they will add to the interest or assist in explanation.

Its extensive circulation and frequency of issue makes it one of the BEST DENTAL ADVERTISING MEDIUMS in the United States.

RATES OF ADVERTISING.

One page, 1 year, $50. Half page, 1 year, $30. Quarter page, or card, 1 year $20.

All advertising bills payable in advance, or at furthest after the issue of the first number containing the advertisement. All transient advertisements to be paid for in advance.

☞ CONTRIBUTIONS SOLICITED.

Address, J. TAFT, or "DENTAL REGISTER," Cincinnati Ohio.

Business Communications to

J. TAFT, Publisher,

No. 117 West Fourth St.

